# Safe Anesthesia for Brugada Syndrome in Pregnancy: Insights From a Retrospective Case Series

**DOI:** 10.7759/cureus.93489

**Published:** 2025-09-29

**Authors:** Ana Rita Fonseca, João Balão, Ana Carneiro, Carla Hipólito, Fernanda Gil

**Affiliations:** 1 Anesthesiology, Unidade Local de Saúde do Alto Ave, Guimarães, PRT; 2 Obstetrics and Gynecology, Unidade Local de Saúde do Alto Ave, Guimarães, PRT

**Keywords:** anesthesia, arrhythmia, brugada syndrome, cardiac monitoring, epidural analgesia, postpartum care, pregnancy

## Abstract

Introduction

Brugada syndrome (BrS) is a cardiac channelopathy associated with sudden cardiac death and presents unique anesthetic challenges during pregnancy due to autonomic fluctuations and sensitivity to sodium channel-blocking agents. This study examines anesthetic strategies and outcomes in pregnant women with BrS.

Methods

We conducted a retrospective review of pregnant patients with confirmed or suspected BrS who delivered at our institution between 2012 and 2023. Data collected included ECG patterns, family history, anesthetic technique and agents, delivery details, and maternal and neonatal outcomes.

Results

Ten women accounted for 21 pregnancy-related events. Regional anesthesia was used in 19 cases: 18 with epidural analgesia (0.2% ropivacaine and opioids) and one spinal with bupivacaine. One patient received remifentanil, and another underwent general anesthesia. No arrhythmic or hemodynamic complications were observed. Mean epidural duration was 5.34 hours. All patients underwent continuous ECG monitoring during labor and for 24 hours postpartum. All neonates had favorable outcomes.

Discussion

Epidural anesthesia appears safe in BrS pregnancies when combined with continuous monitoring and multidisciplinary care. While serum anesthetic concentration is the primary arrhythmogenic risk factor, caution is advised with prolonged infusions due to potential systemic accumulation. The postpartum period remains a high-risk phase requiring extended surveillance.

Conclusion

With appropriate planning and monitoring, neuraxial anesthesia can be safely employed in BrS pregnancies. These findings support the development of tailored peripartum management protocols for this high-risk population.

## Introduction

Brugada syndrome (BrS) is a rare genetic condition that disrupts the heart's electrical activity and increases the risk of sudden cardiac death [1]. It affects 5-20 per 10,000 individuals, predominantly males, in Southeast Asia [1]. Typically manifesting around age 40, BrS is strongly associated with mutations in the *SCN5A* gene, which impairs sodium channel function [2]. This leads to abnormal rhythms, particularly in the right ventricular outflow tract, which predisposes individuals to life-threatening ventricular arrhythmias, such as polymorphic ventricular tachycardia and fibrillation (VT and VF) [3].

Triggers such as fever, certain drugs [4], and shifts in autonomic tone, especially during rest or sleep, can provoke arrhythmias [5]. BrS is now considered a systemic condition, also influencing metabolic and temperature regulation [6]. Pregnancy introduces hormonal and circulatory changes that heighten arrhythmic risk, especially during labor and postpartum, due to increased cardiac output [7].

*SCN5A* mutations may also be linked to sudden infant death syndrome (SIDS), underscoring the role of genetic screening for at-risk families [8-11]. This article reviews BrS in childbirth, presenting a case series from our hospital.

Definition and diagnosis

BrS is primarily caused by *SCN5A* gene mutations that alter sodium channel function in heart cells, producing a characteristic ECG pattern with ST-segment elevation in leads V1-V3 and increasing ventricular arrhythmic risk [11,12]. Diagnosis is based on ECG findings, whether spontaneous or drug-induced, and family history, with *SCN5A* mutations often associated with more severe disease [13].

BrS must be distinguished from the Brugada pattern, which mimics BrS on ECG but can occur transiently due to fever, medications, or electrolyte imbalances. A clinical BrS diagnosis requires a Type 1 ECG pattern plus at least one high-risk feature, such as unexplained syncope, nocturnal agonal breathing, sudden cardiac arrest, family history of early sudden death, or inducible VT/VF during electrophysiology study or documented polymorphic VT/VF [8,14]. Only BrS, not Brugada patterns alone, indicates a higher risk of sudden death.

The validated Shanghai Score System supports diagnosis by integrating ECG, genetics, symptoms, and family history. Scores ≥3.5 indicate definite/probable BrS; 2-3 suggest possible BrS; and <2 are non-diagnostic [15]. This tool may help guide risk assessment and clinical decisions.

Treatment of BrS

Management of BrS focuses on preventing sudden cardiac death and arrhythmias. An implantable cardioverter-defibrillator (ICD) is the most effective option for patients with prior ventricular arrhythmias or cardiac arrest [9,16]. Quinidine may be used to stabilize ion channels, whereas beta-blockers and similar drugs are typically avoided due to their proarrhythmic risk [9]. Lifestyle changes, like avoiding alcohol, fever, and sodium channel-affecting drugs, are also essential [9,16].

Specific risks during pregnancy

Pregnancy introduces physiological and hormonal changes, like increased blood volume and cardiac output, that may increase arrhythmic risk in BrS patients [17]. Effective care requires a multidisciplinary team, including cardiologists, geneticists, obstetricians, and anesthesiologists [7].

Interestingly, some evidence suggests ST-segment elevation may be less pronounced during pregnancy [18]. A large retrospective study found no rise in serious cardiac events but noted a possible increase in spontaneous abortions [19].

Pre-delivery cardiovascular evaluation is critical; ensuring continuous ECG monitoring, antiarrhythmic drugs, and defibrillators readily available are also crucial [7]. While labor induction methods are not contraindicated, the safety of misoprostol is unclear and should be used cautiously [7]. Oxytocin, although theoretically proarrhythmic (by triggering intracellular calcium release), is generally considered safe [7,20], whereas ergometrine is best avoided, as expert consensus and case reports identify it as a potentially proarrhythmic agent in patients with BrS [7].

Anesthetic plans must be individualized, as both regional and general techniques present distinct risks.

Regional anesthesia/analgesia

Local anesthetics block sodium channels and should be avoided in BrS when possible. If needed, lidocaine with epinephrine is preferred for its Class 1b antiarrhythmic properties [13].

Epidural anesthesia allows controlled dosing and a gradual onset, which can help reduce sudden cardiovascular shifts; however, its use may result in higher cumulative doses and increased plasma concentrations, potentially raising arrhythmic risk. Although bupivacaine has known arrhythmogenic potential [21], some case reports describe its safe use in BrS patients [20,22,23].

General anesthesia

Choice of agents is critical. While propofol has been associated with ST-segment changes in BrS [24], studies have shown that single bolus or continuous doses for induction were not associated with increased arrhythmic risk [25,26]. Etomidate is often considered more electrophysiologically stable, though at least one study found no significant difference between the two agents [26].

Postpartum

The postpartum period carries a high arrhythmic risk in BrS patients due to sudden hemodynamic and hormonal shifts. Continuous ECG monitoring for at least 24 hours is strongly recommended to allow early detection and prompt management of complications [7].

Our study: background

The city of Guimarães, where our hospital is located, has a notably high prevalence of BrS. Consequently, pregnant women with BrS frequently require specialized care in our emergency department. Due to the limited data on BrS in pregnancy, we conducted a retrospective study examining peripartum management and outcomes in this population.

## Materials and methods

We conducted a retrospective chart review of all women with confirmed or suspected BrS who were admitted for childbirth or miscarriage management between January 2012 and December 2023 at Unidade Local de Saúde do Alto Ave, Guimarães, Portugal. The study was approved by the institutional review board (approval number 115/125 CAF), and patient confidentiality was maintained in accordance with the principles outlined in the Declaration of Helsinki.

Inclusion and exclusion criteria

BrS was identified based on the following criteria: a diagnostic electrocardiographic (ECG) type 1 pattern, either spontaneous or drug-induced, and clinical factors such as a positive family history or previous cardiovascular events or symptoms. The inclusion criteria comprised all women with a confirmed or suspected diagnosis of BrS who presented for labor, delivery, or miscarriage management during the study period.

Exclusion criteria included women without any available or diagnostic cardiac evaluations (e.g., missing or normal ECGs, no BrS features) and those who were later found to have negative confirmatory testing for BrS.

**Figure 1:**
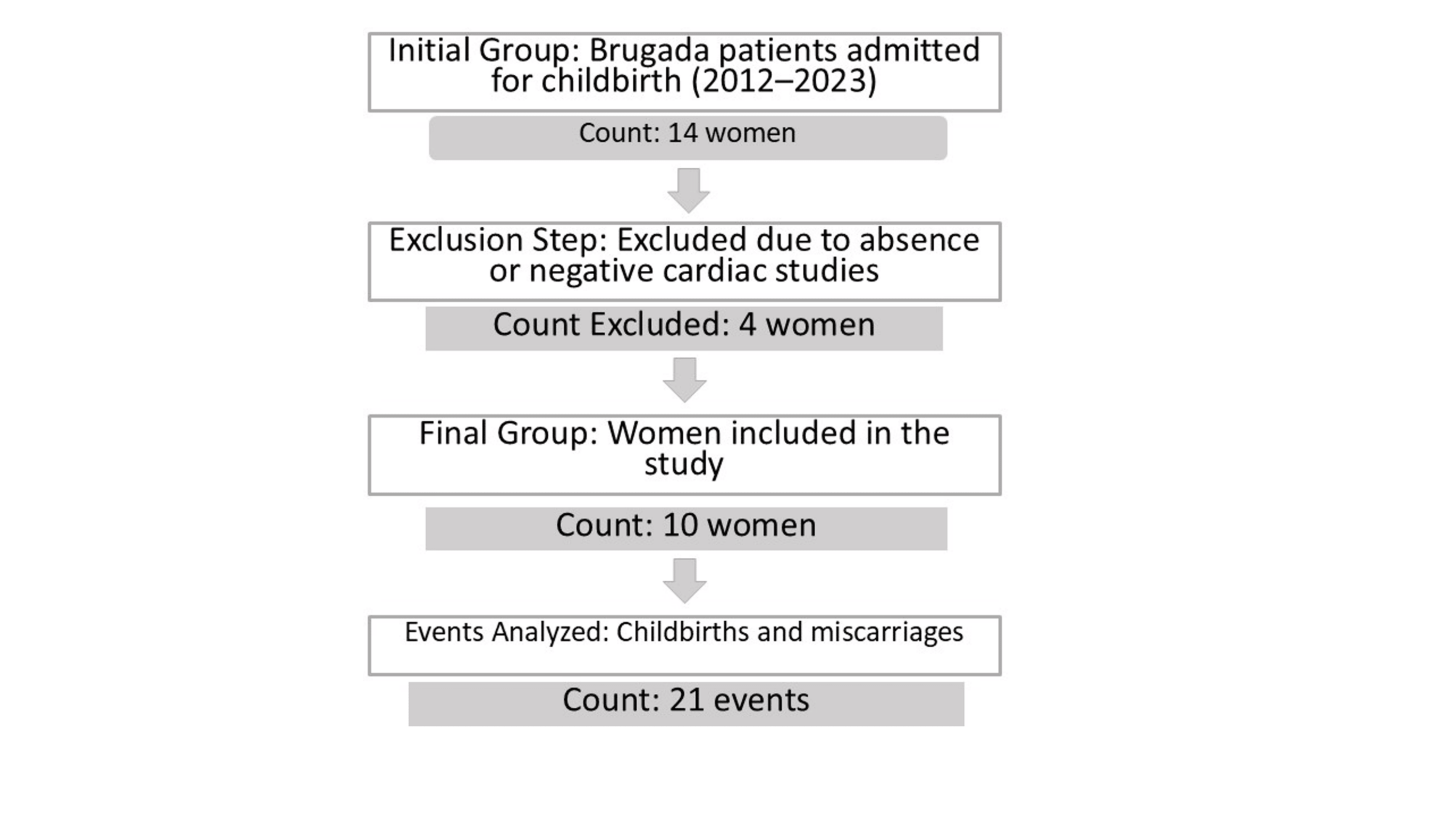
Study Flowchart

Fourteen women were initially identified; four were excluded based on the above criteria, resulting in a final sample of 10 women and 21 obstetric events (including multiple deliveries and miscarriages). The selection process is illustrated in Figure 1.

Data collection

Data were collected from both electronic and paper-based health records, including obstetric databases and anesthetic charts. A standardized data extraction form was used to ensure consistency in data retrieval and minimize variability between records.

Variables collected

The following variables were extracted and categorized as either categorical or continuous. Categorical variables included the presence of an ICD, use of cardiac or other chronic medications, type of Brugada ECG pattern (spontaneous or drug-induced), family history of BrS, mode of delivery (vaginal or cesarean), type of anesthesia (epidural, spinal, general, or remifentanil), and whether ropivacaine was used. Continuous variables included patient age, body mass index (BMI), duration of labor (in hours), gestational age at delivery, ropivacaine dosage (measured in mg or mL, depending on documentation), and length of hospital stay (in days).

Statistical analysis

Data were analyzed using Microsoft Excel (Microsoft Corporation, Redmond, Washington). All analyses were descriptive in nature, given the small sample size and retrospective design. Categorical variables are presented as absolute frequencies and percentages. Continuous variables were summarized only using means. No formal assessment of data distribution or variability was performed due to the small sample size.

## Results

The women included in this study were aged between 24 and 37 years at the time of hospital admission. The characterization of the 10 women ultimately included in our analysis is presented in Table 1.

**Table 1:** Population Characterization BrS: Brugada Syndrome; ECG: Electrocardiogram; ICD: Implantable Cardioverter-Defibrillator

Patient Characteristics	N	%
ICD	1	10%
Cardiology medication	0	0%
No other medication	10	100%
ECG (Induced type 1)	9	90%
ECG (Spontaneous type 1)	1	10%
Family history of Brugada syndrome	9	90%

The information relative to the 21 procedures included in our analysis is presented in Tables 2, 3.

**Table 2:** Type of Delivery

Type of Delivery	N	%
Vaginal (Eutocic)	12	57%
Vaginal (Dystocic)	3	14%
Cesarean Section	5	24%
Miscarriage	1	5%
Total	21	100%

**Table 3:** Type of Anesthesia

Type of Anesthesia	N	%
Balanced General Anesthesia	1	5%
Remifentanil	1	5%
Regional Anesthesia (Spinal Anesthesia)	1	5%
Regional Anesthesia (Epidural)	18	85%
Total	21	100%

**Figure 2:**
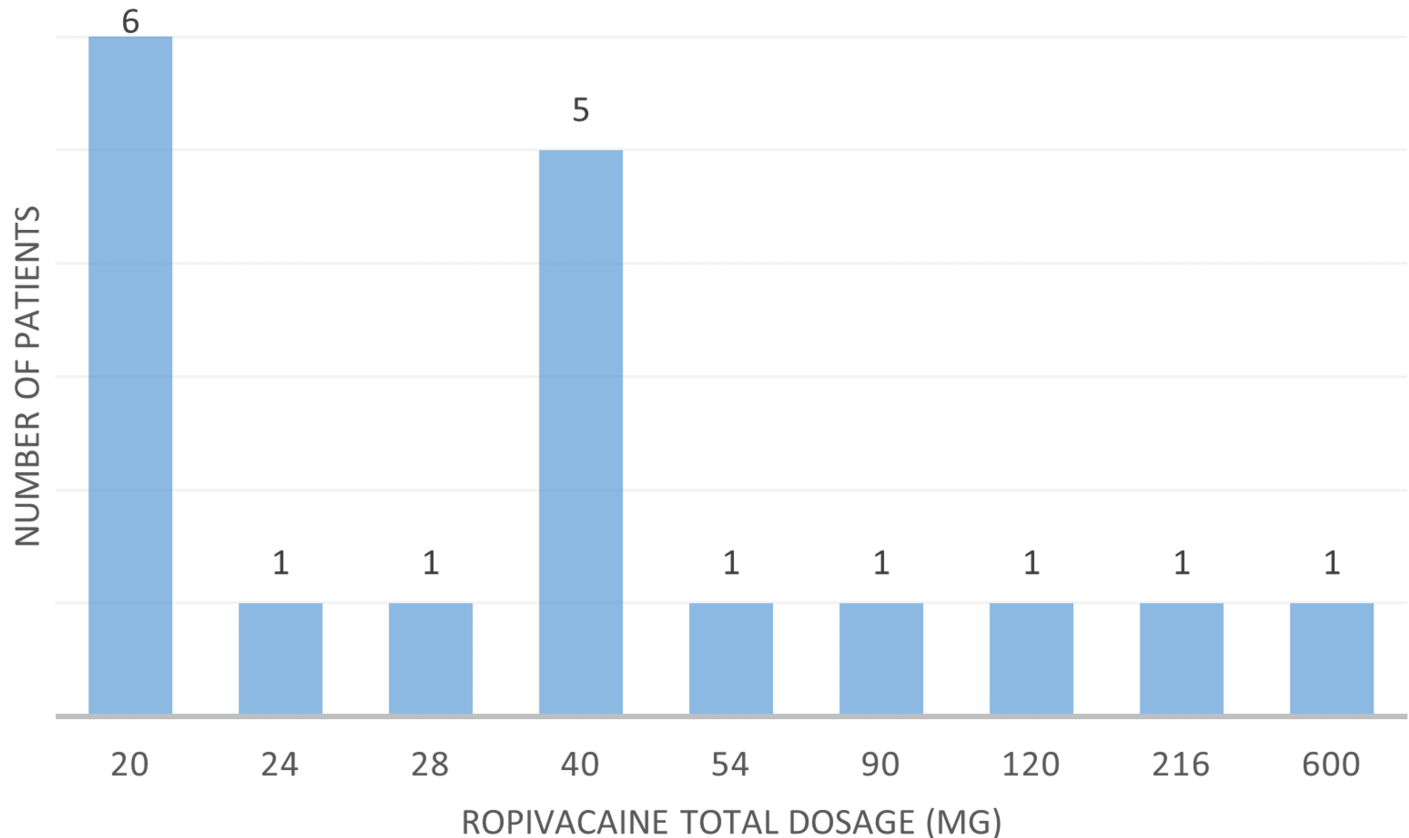
Ropivacaine Dosage

Labor analgesia lasted between 30 minutes and 48 hours (mean 5.34 hours), with no complications reported. Of the 21 cases, 19 received regional techniques: 18 epidurals (0.2% ropivacaine with fentanyl or sufentanil for labor; 0.75% ropivacaine for cesarean sections, except in one case where 2% lidocaine was used) and one spinal (0.5% hyperbaric bupivacaine, 7 mg). One patient received remifentanil analgesia, and another underwent cesarean section under general anesthesia with rapid sequence induction using propofol (2 mg/kg), rocuronium (1.2 mg/kg), and sevoflurane to maintain a BIS between 40 and 60. Fentanyl (2 µg/kg) was administered after delivery, and oxytocin was given as a 10-unit bolus followed by an infusion of 7.5 units per hour for two hours. The distribution of ropivacaine dosages is illustrated in Figure 2.

All women underwent continuous ECG monitoring during childbirth, with additional monitoring of temperature, oxygen saturation, capillary glucose, and, when applicable, bispectral index during general anesthesia. An external defibrillator and resuscitation drugs were also kept immediately available as part of contingency planning for potential malignant arrhythmias.

Postpartum, all patients underwent 24-hour monitoring in a high-dependency unit before transfer to the general ward. Hospital stays averaged 2.87 days, with no recorded complications. Most women remained under cardiology follow-up, and no further events occurred. Children were referred for pediatric cardiology evaluation.

## Discussion

Managing pregnancy in women with BrS is complex due to the physiological adaptations of gestation, potential arrhythmogenic triggers during labor, and the effects of anesthetic agents on cardiac ion channels. Our study offers valuable insights into peripartum management, emphasizing anesthetic strategies, delivery planning, and postpartum surveillance.

Anesthetic considerations and safety

Our data suggest that regional anesthesia, particularly epidural analgesia with ropivacaine and opioids, can be administered safely in BrS patients when accompanied by continuous ECG monitoring. Although bupivacaine is often avoided due to its potential to block sodium channels and prolong repolarization [23], it has been used without complications [20,22,27], consistent with other reports describing cautious local anesthetic use.

Other studies also report safe use of ropivacaine [28,29]. However, current evidence suggests that systemic plasma concentrations, determined by factors such as potency, dose, infusion rate, and pharmacokinetics, are a key factor in arrhythmic risk [1,12]. As prolonged infusions can lead to accumulation, caution is advised regarding total anesthetic dose, especially in prolonged labor. Continuous ECG monitoring remains fundamental for detecting and treating arrhythmias early, supported by access to antiarrhythmics and defibrillators.

Labor and delivery outcomes

The mode of delivery did not influence maternal outcomes in our cohort, echoing existing literature. Both vaginal and cesarean births were safely performed, reinforcing that BrS is not in itself an indication for cesarean section. Although some studies associate BrS with higher rates of spontaneous abortion, we observed no such pattern. The attenuation of ST-segment elevation during pregnancy [18] may reflect temporary hormonal protection, although this does not negate the importance of close postpartum monitoring, when arrhythmic risk rises due to hemodynamic and endocrine changes.

Clinical implications

Our findings reinforce several key recommendations for the management of BrS during pregnancy. Epidural analgesia may be safely administered when appropriate agents are selected and dosing is carefully monitored. Continuous electrocardiographic monitoring during labor and for at least 24 hours postpartum is crucial for promptly detecting arrhythmias. Anesthetic agents should be chosen with caution, favoring those with lower arrhythmogenic potential, such as ropivacaine, lidocaine with epinephrine, and etomidate. To better guide the selection of anesthetic agents in patients with BrS, Table 4 categorizes commonly used drugs based on their proarrhythmic risk, according to available evidence [30].

**Table 4:** Risk of Commonly Used Drugs in Patients With Brugada Syndrome *It is recommended to administer muscle relaxant reversals slowly, as anticholinergics can cause tachycardia and neostigmine can cause ST elevation. **Isoproterenol reduces ST-segment elevation and suppresses arrhythmias. ***Due to the risk of propofol perfusion syndrome and consequent changes in ion channels. Table created by the authors. This classification is based on data from Postema et al. [30], as summarized on www.brugadadrugs.org.

Category	Drug
Safe drugs (no evidence of adverse effects in patients with Brugada syndrome)	Benzodiazepines
	Propofol (bolus)
	All halogenated
	Muscle Relaxants and their antagonists*
	Dexamethasone
	Ondansetron
	Droperidol
	Ketorolac
	Local Anesthetics (except Bupivacaine)
	Barbiturates
	Atropine
	Glycopyrrolate
	Dopamine
	Ephedrine
	Phenylephrine
	Isoproterenol/Isoprenaline**
Drugs to use with caution	Ketamine (if in high doses)
	Nitrogen protoxide (for its effect on the QT interval)
	ß-blockers and α agonists (may cause QT elevation)
Drugs to avoid	Propofol (infusion)***
	Bupivacaine
	Metoclopramide
	Phenytoin
	Tramadol
	Verapamil
	Carbamazepine
	Tricyclic antidepressants
	Lithium
Contraindicated drugs	Flecainide
	Procainamide
	Amiodarone
Drugs without evidence	Nifedipine
	Nitroglycerin
	Diltiazem

A multidisciplinary team approach involving obstetrics, anesthesiology, and cardiology is vital for optimizing maternal and fetal outcomes. Given the hereditary nature of BrS, neonatal cardiology follow-up and genetic screening should also be considered.

Limitations and future directions

Although our study offers important insights into the peripartum management of BrS, its retrospective nature and small sample size limit broader applicability. Larger, multicenter studies are needed to confirm these findings and inform clearer guidelines. Future research should also explore how hormonal changes during pregnancy influence cardiac electrophysiology in BrS, potentially explaining the observed reduction in arrhythmic risk in some women.

## Conclusions

Our results suggest that with individualized anesthetic planning, vigilant monitoring, and multidisciplinary care, pregnant women with BrS can safely undergo labor and delivery. Epidural analgesia appears safe when used with caution, and anesthetic choices should prioritize agents with lower arrhythmic potential.

Despite favorable maternal and neonatal outcomes, the postpartum period requires close surveillance due to elevated arrhythmic risk. This study contributes to the evolving understanding of BrS in pregnancy and highlights the importance of continued cardiology follow-up and genetic evaluation. Broader studies are essential to refine clinical protocols and support long-term care strategies for affected mothers and their children.
